# Using biomarkers to predict progression from clinically isolated syndrome to multiple sclerosis

**DOI:** 10.1186/2043-9113-3-18

**Published:** 2013-10-03

**Authors:** John T Tossberg, Philip S Crooke, Melodie A Henderson, Subramaniam Sriram, Davit Mrelashvili, Saskia Vosslamber, Cor L Verweij, Nancy J Olsen, Thomas M Aune

**Affiliations:** 1 Research Department, ArthroChip, LLC, Franklin, TN, USA; 2 Department of Mathematics, Vanderbilt University, Nashville, TN, USA; 3 Department of Medicine, Vanderbilt University School of Medicine, MCN T3219, 1161 21st Avenue South, Nashville, TN, 37232-2681, USA; 4 Department of Neurology, Vanderbilt University School of Medicine, Nashville, TN, USA; 5 Department of Neurology, University of South Carolina, Columbia, SC, USA; 6 Department of Pathology, VU University Medical Center, Amsterdam, The Netherlands; 7 Department of Medicine, Penn State Hershey Medical Center, Hershey, PA, USA; 8 Department of Pathology, Microbiology and Immunology, Vanderbilt University School of Medicine, Nashville, TN, USA

**Keywords:** Genomics, Multiple sclerosis, Disease prediction, Diagnosis

## Abstract

**Background:**

Detection of brain lesions disseminated in space and time by magnetic resonance imaging remains a cornerstone for the diagnosis of clinically definite multiple sclerosis. We have sought to determine if gene expression biomarkers could contribute to the clinical diagnosis of multiple sclerosis.

**Methods:**

We employed expression levels of 30 genes in blood from 199 subjects with multiple sclerosis, 203 subjects with other neurologic disorders, and 114 healthy control subjects to train ratioscore and support vector machine algorithms. Blood samples were obtained from 46 subjects coincident with clinically isolated syndrome who progressed to clinically definite multiple sclerosis determined by conventional methods. Gene expression levels from these subjects were inputted into ratioscore and support vector machine algorithms to determine if these methods also predicted that these subjects would develop multiple sclerosis. Standard calculations of sensitivity and specificity were employed to determine accuracy of these predictions.

**Results:**

Our results demonstrate that ratioscore and support vector machine methods employing input gene transcript levels in blood can accurately identify subjects with clinically isolated syndrome that will progress to multiple sclerosis.

**Conclusions:**

We conclude these approaches may be useful to predict progression from clinically isolated syndrome to multiple sclerosis.

## Background

Diagnosis of multiple sclerosis [MS] rests on clinical symptoms and examination as outlined in the revised McDonald’s criteria supported by appropriate magnetic resonance imaging findings or other laboratory tests such as detection of oligoclonal bands in cerebrospinal fluid and evoked potential testing [1-7]. Clinically isolated syndrome (CIS) is a first neurologic episode lasting at least 24 hours possibly caused by focal inflammation or demyelination [8,9]. Approximately 10,000-15,000 new diagnoses of MS are made in the United States each year [10]. Approximately 2–3 times that number experience a CIS each year indicating that a far greater number of subjects experience a CIS than develop MS [11-14]. Costs to healthcare of determining if a subject with a CIS will develop MS are significant considering the cost of MRI and additional testing performed and the fact that many more subjects develop CIS than MS.

Presence of abnormal MRI findings and detection of oligoclonal bands in the cerebrospinal fluid in an individual at the time of CIS increase the likelihood of an eventual diagnosis of MS. However, these findings do not guarantee an eventual diagnosis of MS nor do their absence preclude a diagnosis of MS. We have considered that measuring gene transcript patterns in blood may provide a means to develop tests with the ability to exclude the diagnosis of a given disease, such as MS, or to establish a diagnosis of MS, and have performed studies to identify gene expression patterns that distinguish subjects with MS from a) healthy control subjects, b) subjects with inflammatory neurologic conditions distinct from MS (other inflammatory neurologic conditions, OND-I), e.g. transverse myelitis [1], neuromyelitis optica (NMO) and c) subjects with other non-inflammatory neurologic conditions (OND-NI) [15,16]. We have also applied this approach to gastro-intestinal diseases and have found it possible to discriminate between irritable bowel syndrome and inflammatory bowel disease, two conditions with similar clinical presentations, and to discriminate between the two most frequent and related forms of inflammatory bowel disease, ulcerative colitis and Crohn’s disease, thus demonstrating the general utility of our approach [17].

A limitation to these studies is that subjects included in these analyses do not completely represent patients in the general population in whom these tests may be performed. Presumably, tests would be performed on subjects who do not yet have a clinical diagnosis of a given disease. To address this limitation, we decided to examine subjects at the time they experience CIS who acquire a diagnosis of MS in the future using established criteria. We applied two independent analytic methods, a ratioscore algorithm we previously developed and support vector machines. Our results demonstrate that these methods predict future conversion to MS with a high degree of specificity.

## Methods

### Human subjects

Blood samples in PAXgene tubes were obtained from CTRL, MS, OND-I and OND-NI subjects. Samples were also obtained from subjects with CIS at the time of the blood draw. All of these subjects have gone on to develop MS according to the McDonald’s criteria for the diagnosis of MS. Age, race and gender were not statistically different among the different study groups. Time of blood draw, for example, morning/afternoon clinics, was also not statistically significant among the different study groups. Relevant institutional review board approval was obtained from all participating sites.

### Transcript determinations

Total RNA purification, cDNA synthesis, and analysis using a 384-well Taqman Low Density Array (TLDA) were as previously described (Additional file 1: Figure S1) [16,17]. Patient diagnosis was blinded for all experimental procedures. Relative expression levels were determined directly from the observed threshold cycle (C_Τ_). Linear expression levels were determined using the formula, 2^(40-CΤ)^.

### Ratioscore and support vector machine algorithms

The identification of the gene expression ratios and permutation testing strategy employed to identify discriminatory combinations of ratios to create the ratioscore have been previously described.^16^ Briefly, all possible gene-expression ratios were computed. Ratios in which the greatest number of subjects in case groups possessed a ratio value greater than the highest ratio value in the control group were saved. We performed permutation testing by randomly selecting 80% of the control group to compare with the case group and repeating this process 200 times producing 200 subsets of ratios. From these subsets of ratios, we identified the smallest number of ratios to identify the ratioscore with maximum separation between case groups and control groups. For example, we compared MS versus CTRL, MS versus OND, *etc*. Each comparison produced a unique set of ratios that were used to define the ratioscore algorithm for that pairing of the case–control groups.

A support vector machine (SVM) was created from each set of ratioscores using LS-SVMLab software (http://www.esat.kuleuven.be/sista/lssvmab). For example, the gene-expression ratios from the MS versus CTRL were used to create a SVM for this type of comparison. The SVM was trained with *L-fold cross-validation* using 60% of the data. In this type of training a certain fraction of the training set was omitted from training and the remaining portion of the partial training set was used to estimate the parameters in the SVM. Once the SVM was trained, the SVM was applied to the total data set. Numbers of correct and incorrect classifications were tabulated for total sets (training and validation), training sets and validation sets. As expected, the overall accuracy in the training sets was greater than overall accuracy of the validation sets.

### Analysis of CIS➔MS subject data

Gene expression ratio data obtained from CIS➔MS cohort samples were determined and applied to the ratioscore or SVM defined by the independent training cross-comparisons, e.g. CTRL versus MS, OND versus MS. New subjects were classified into their respective category based upon their profile of gene expression ratios.

## Results and discussion

### Study cohorts

A total of 562 subjects were included in the study: 199 with clinically definite MS, 203 with OND segregated into 84 OND-I subjects and 119 OND-NI subjects, 114 healthy control subjects and 46 subjects whose blood sample was obtained at the time of their CIS but who now have progressed to clinically definite MS, CIS➔MS (Table 1). MS patients were divided into two additional categories: those at their initial diagnosis of MS but before initiation of therapies; MS-naïve, and those ≥1 year after diagnosis of MS and on different therapies; MS-established. The overall laboratory and analytic processes are summarized in Additional file 1: Figure S1.

**Table 1:** Demographic characteristics of the different subject populations

	**#**	**Age**	P*	**Gender**	***P***	**Ethnicity**	***P***
				**(% F)**		(%, C/AA/As/H)**	
**MS**	**199**	**43 ± 10**	**NS**	**76**	**NS**	**80/20/0/0**	**NS**
**OND-I**	**84**	**46 ± 10**	**NS**	**68**	**NS**	**67/33/0/0**	**NS**
**OND-NI**	**119**	**46 ± 10**	**NS**	**67**	**NS**	**68/26/3/1**	**NS**
**CTRL**	**114**	**41 ± 11**		**77**		**71/22/3/3**	
**CIS ➔MS**	**46**	**35 ± 6**	**NS**	**72**	**NS**	**82/14/4/0**	**NS**

*MS* MS-treatment naïve (N = 85), MS with established disease on medications (N = 114), *OND-I* other inflammatory neurologic disorders, acute disseminated encephalomyelitis (N = 4), Bell’s Palsy (N = 3), CNS lupus (N = 2), Guillaine Barre (N = 4), Myasthenia Gravis (N = 3), Neuromyelitis optica (N = 26), Optic neuritis (N = 1), Transverse myelitis (N = 41), *OND-NI* other non-inflammatory neurologic disorders, Alzheimer’s (N = 6), cerebral ataxia (N = 2), cerebral bleed (N = 2), cervical radiculopathy (N = 6), drug-induced movement disorder (N = 1), dystonia (N = 1), epilepsy (N = 4), essential tremor (N = 9), Huntington’s disease (N = 1), hydrocephalus (N = 1), median neuropathy (N = 2), meningioma (N = 1), migraine (N = 30), Parkinsons (N = 23), peripheral neuropathy (N = 1), pseudotumor (N = 3), restless leg syndrome (N = 6), seizures (N = 9), stroke (N = 10), *CIS***➔***MS* subjects who had clinically isolated syndrome at the time of the blood draw who have developed clinically definite MS.

U.S sites: TN, MA, MD, NY, SC, AZ, TX, CA, samples from sites in MS, MD, NY, AZ, and CA were obtained through the Accelerated Cure Project, European sites: Denmark, Netherlands

*P calculated by Student’s T-test [18] or Fisher’s exact test, NS: *P* > 0.05, calculated relative to CTRL.

**C Caucasian, *AA* African American, *As* Asian, *H* Hispanic.

### Transcript profiles

**Figure 1:**
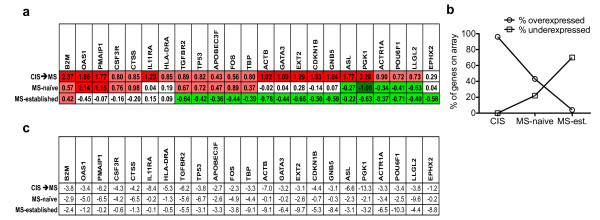
**Gene-expression profiles in subjects with CIS, MS-naïve or MS-established. (a)** Expression levels of 23 target genes were determined by quantitative reverse-transcription PCR and normalized to expression of GAPDH. Results are expressed as the ratio of the expression level of the indicated genes in the disease cohort relative to the CTRL cohort, log2. Genes are identified that showed statistically significant (P < 0.05 after Bonferroni’s correction for multiple testing) increased (red boxes) or decreased (green boxes) expression. Numerical expression ratios, log2, of the test/CTRL cohorts are displayed within the boxes. **(b)** Cumulative percentage of over- and under-expressed genes in each disease cohort relative to CTRL. **(c)** Statistical significance of the expression level of each target gene between each disease cohort and CTRL was determined using Student’s T test. P values are expressed as log_10_.

We determined the transcript level in blood for each target gene relative to *GAPDH* in the three study groups, CIS➔MS, MS-naïve, MS-established and the CTRL group using TLDA plates. Target genes were selected from previous microarray studies [19-21]. Inclusion of the specific gene targets was based upon the following criteria: (a) previous studies demonstrating differential expression among control and multiple autoimmune disease cohorts, (b) protein products possess known pro- or anti-inflammatory functions, (c) expression levels change in response to pro-inflammatory stimuli (cytokines), and/or (d) protein products have known roles in cell cycle progression and/or apoptosis. The ratio, log_2_, of the expression level of each gene in each study group was calculated relative to CTRL and results are presented in a heatmap, over-expressed: red, under-expressed: green. Numerical ratios, log_2_, are displayed within each box (Figure 1a). Transcript profiles in the three study groups, CIS➔MS, MS-naïve, and MS-established were highly dynamic. In the CIS➔MS cohort, most genes were significantly over-expressed relative to CTRL. In contrast, the majority of target genes were significantly under-expressed in the MS-established cohort. The MS-naïve cohort was intermediate with an almost equal number of over- and under-expressed genes (Figure 1b). Using the student’s T test, we determined P-values, log_10_, comparing each study group cohort to the CTRL cohort (Figure 1c). Differences in transcript levels of many genes were highly significant among the different study groups. Of note, the P-value, log_10_, for *PGK1* expression between the CIS➔MS cohort and CTRL cohort was -13.3. Similarly, expression differences of *LLGL2* was most significant in the MS-naïve cohort, log_10_ = -9.6 and expression differences of *POU6F1* was most significant in the MS-established cohort, log_10_ = 10.3. One interpretation of these results is that each subject within each of these three disease cohorts, CIS➔MS, MS-naïve, and MS-established, has a very similar target gene transcript profile suggesting that each is mediated by a common underlying molecular pathway(s) or event(s). Even though this is a cross-sectional rather than a longitudinal study, a second interpretation of these results is that target gene transcript profiles are highly dynamic as a subject progresses from CIS to clinically definite MS to MS disease of some duration.

#### Ratioscore algorithm

**Figure 2:**
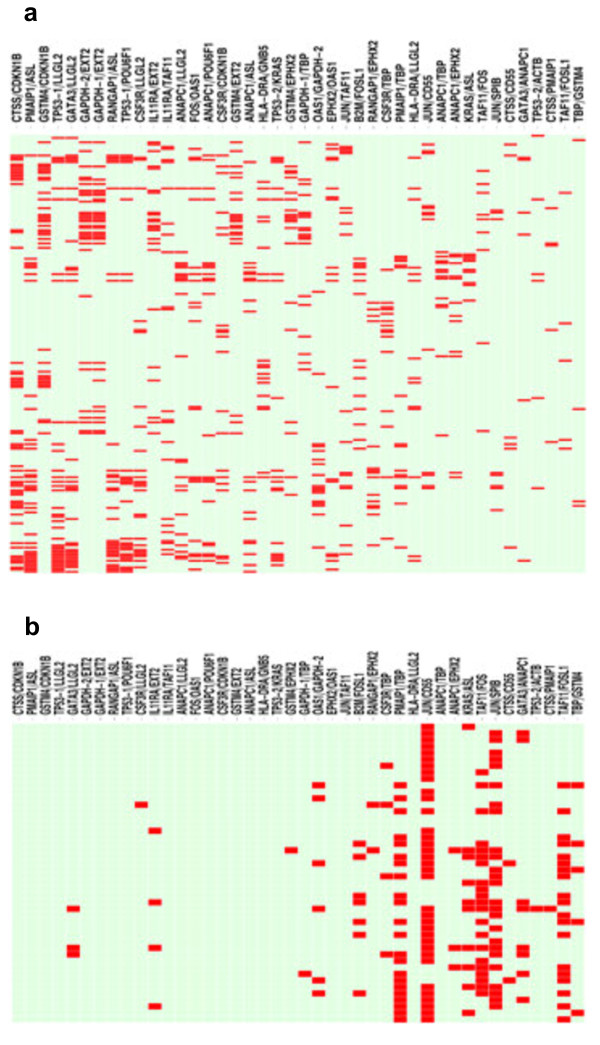
**Heatmap of results from the ratioscore algorithm for the MS: CTRL comparison. (a)** Training set: Columns represent individual ratios. Rows represent individual subjects within the MS cohort. Red in the heatmap denotes individual subjects with the value of the individual ratio greater than the value of the ratio in all subjects within the CTRL cohort. Green denotes individual subjects with the value of the individual ratio less than or equal to the highest ratio value in all subjects within the CTRL cohort. **(b)** Results from inputting independent CIS➔MS subjects into the ratioscore algorithm.

We used the previously described ratioscore method to compute all gene expression ratios and permutation testing to identify the set best able to discriminate the MS cohort, naïve and established combined, from the CTRL cohort [16]. We generated a heatmap to depict which ratios (columns) were positive for each MS subject (rows; red bars indicate positive score) (Figure 2a). One or more positive ratios produce a score ≥ 1 making a subject positive for the indicated disease, in this case, MS. A total of 179 of 199 MS subjects (90%) were assigned to the MS category using the ratioscore method and 100% of CTRL subjects were excluded from the MS category. Using these gene expression ratios, we input data from the CIS➔MS cohort to determine if these subjects would fall into the MS or CTRL category. As above, we constructed a heatmap to depict which ratios (columns) were positive in each CIS➔MS subject (rows). A total of 44 of 46 CIS➔MS subjects (96%) were assigned to the MS category using the ratioscore defined for MS (Figure 2b).

**Figure 3:**
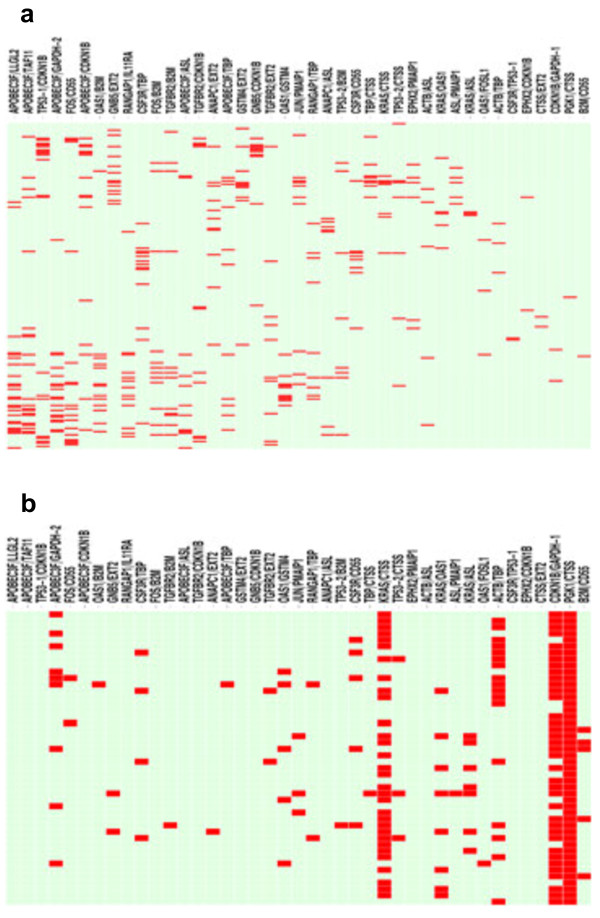
**Heatmap of results from the ratioscore algorithm for the MS: OND comparison. (a)** Ratios define the ratioscore discriminating MS from OND. Columns represent individual ratios. Rows represent individual subjects within the MS cohort. Red in the heatmap denotes individual subjects with the value of the individual ratio greater than the value of the ratio in all subjects within the CTRL cohort. Green denotes individual subjects with the value of the individual ratio less than or equal to the highest ratio value in all subjects within the CTRL cohort. **(b)** Results from inputting independent CIS➔MS subjects into the ratioscore algorithm.

Using a similar approach, we employed the ratioscore algorithm to compute ratios to discriminate MS, combined MS-naïve and MS-established from OND. As above, we generated a heatmap to depict which ratios (columns) were positive for each MS subject (rows, red bars indicate positive score) (Figure 3a). A total of 140 of 199 MS subjects (70%) were assigned to the MS category using the ratioscore method and 203 of 203 (100%) of OND subjects were excluded from the MS category. As above, using these gene expression ratios, we input data from the CIS➔MS cohort to determine if these subjects would fall into the MS or CTRL category. We constructed a similar heatmap to depict which ratios (columns) were positive in each CIS➔MS subject (rows). A total of 46 of 46 CIS➔MS subjects (100%) fell into the MS category using the ratioscore method (Figure 3b).

Our rationale for performing this two-tier analysis rather than combining the CTRL and OND subjects into one cohort was that previous studies demonstrated that accuracy was severely compromised. To confirm that this was the case in this analysis we compared the MS cohort to the combined CTRL plus OND cohort and inputted these data into the ratioscore algorithm. As expected, overall ability to discriminate MS from this combined cohort was compromised. Only 58% of MS subjects were assigned to the MS category while 100% of subjects in the combined CTRL plus OND cohort were excluded from the MS category (Additional file 2: Figure S2A). When we input data from the CIS➔MS cohort, only 28 of 46 subjects (61%) were categorized as MS (Additional file 2: Figure S2B). Thus, overall accuracy of the ratioscore method was much improved by performing two tiers of analyses, first MS versus CTRL, then MS versus OND.

We also sub-divided the OND cohort into OND-I and OND-NI (Table 1) and repeated the ratioscore algorithm to assess how well these sub-groups could be distinguished from MS (Additional file 3: Figure S3A & B). In the OND-I versus MS comparison, 90% of MS subjects were assigned to the MS class and 100% of OND-I subjects were excluded from the MS class. When we input data from the CIS➔MS cohort, 46 of 46 subjects (100%) were categorized as MS. In the OND-NI versus MS comparison, 86% of MS subjects were assigned to the MS class and 100% of OND-NI subjects were excluded from the MS class. When we input data from the CIS➔MS cohort, 46 of 46 subjects (100%) were categorized as MS. We conclude that this further subdivision of OND subjects produces only limited improvement in overall accuracy.

#### Accuracy of ratioscore and SVM methods

We also trained a SVM with ratios identified by the ratioscore method using 60% of CTRL subjects and 60% of cases (see Methods). We validated the SVM with the remaining 40% of CTRLs and cases. Subjects within the CIS➔MS cohort were input into the SVM to ascertain if the SVM would identify them as controls or cases. New SVMs were created using 60% of OND, OND-NI, and OND-I cohorts as controls, respectively and 60% of MS subjects as the case cohort. SVMs were validated with the remaining 40% of the respective control cohort and remaining 40% of the case cohort [22]. As above, subjects within the CIS➔MS cohort were input into each SVM to ascertain if the SVM would identify them as controls or cases. Results from the SVM method were compared to results from the ratioscore method by calculating sensitivity and specificity (Table 2). Overall, ratioscore and SVM produced comparable sensitivity and specificity in control : case comparisons. More relevant, subjects within the CIS➔MS cohort were identified as MS by both methods with a high degree of specificity. Thus, we propose this tiered approach, MS : CTRL then MS : OND, could be employed to predict if a subject with CIS will develop MS with a reasonable level of overall accuracy.

**Table 2:** Sensitivity and specificity of ratioscore and SVM methods

			**Ratioscore**		**SVM**	
	**Control**	**Case**	**Sensitivity**	**Specificity**	**Sensitivity**	**Specificity**
#1	CONTROL	MS	0.87	1.00	0.87	0.97
	CONTROL	CIS **➔** MS	0.96		0.95	
#2	OND	MS	0.70	1.00	0.82	0.78
	OND	CIS **➔** MS	1.00		1.00	
#3	OND-NI	MS	0.86	1.00	0.84	0.94
	OND-NI	CIS **➔** MS	1.00		1.00	
#4	OND-I	MS	0.90	1.00	0.77	0.93
	OND-I	CIS **➔** MS	1.00		0.98	

Optimum ratios for the ratioscore method were from Figures 2, 3 and Additional file 3: Figure S3. CIS **➔** MS subject data were inputted and scores computed. For the SVM, 60% of controls and cases were randomly selected for the training set and 40% were used for the validation set. Sensitivity and specificity were calculated for the combined sets. These results defined the SVM. CIS **➔** MS subject data were applied to the SVM and subjects received a score of 0 if assigned to the CONTROL cohort or 1 if assigned to the CASE cohort. Sensitivity was calculated from this output.

Sensitivity = # true positives/(# true positives + # false negatives).

Specificity = # true negatives/(# true negatives + # false positives).

To summarize, overall transcript profiles in the CIS➔MS, MS-naïve, and MS-established were markedly different and we suggest that these dynamic transitions may reflect different pathogenic states of MS or progression of MS. Thus, we suggest that this gene expression analysis could also be used to classify different stages of MS in an individual. In addition, studying the molecular origins of the robust transcript signature in CIS➔MS subjects may produce insights into the origins of MS. In spite of the differences in overall transcript profiles in these three subject groups, ratioscore and SVM methods were able to assign CIS➔MS subjects to the MS category with a high degree of accuracy. This is due, in part, to the fact that the ratioscore method does not require that all subjects within these three cohorts representing three distinct stages of disease progression possess identical gene expression signatures. In contrast, many other standard methods of analysis of gene expression signatures are dependent upon identification of overall differences between or among groups.

A limitation to this study is that we did not include subjects with an initial CIS that did not develop MS. Our rationale for not including this parameter is three-fold. First, there is not a uniform clinical definition of CIS. Second, subjects with a CIS may or may not have MRI findings indicating inflammation or demyelination and the probability that a subject with CIS will develop MS is greater if MRI lesions are also detected. Third, with our current knowledge, it is uncertain if it is experimentally possible to absolutely conclude that a person with CIS will not develop MS. In fact, the period of time between an initial CIS and diagnosis of clinically definite MS is quite variable and can exceed 5 years.

## Abbreviations

CIS: Clinical isolated syndrome; CTRL: Control; MRI: Magnetic resonance imaging; MS: Multiple sclerosis; NMO: Neuromyelitis optica; OND: Other neurologic disorders; OND-I: Other inflammatory neurologic disorders; OND-NI: Other non-inflammatory neurologic disorders; SVM: Support vector machines; TLDA: Taqman low density array; TM: Transverse myelitis.

## Competing interests

TMA and NJO are co-owners of ArthroChip. Other authors claim no competing interests.

## Authors’ contributions

JTT, PSC, NJO and TMA conceived and designed the study. JTT produced the data. PSC designed algorithms and other analytic methods with assistance from TMA. PSC and TMA performed data analysis. MAA managed databases and sample acquisition and inventory. SS, DM, SV, and CLV provided critical patient samples and clinical information. TMA and PSC wrote the manuscript with input from JTT, MAA, SS, DM, SV, CLV, and NJO. All authors read and approved the final manuscript.

## Supplementary Material

Additional file 1: Figure S1 Flow chart describing sample collection and processing, data generation, and methods of data analysis. Click here for file

Additional file 2: Figure S2 **a**. Ability of the ratioscore method to discriminate between MS and combined CTRL plus OND subjects. Columns represent individual ratios. Rows represent individual subjects within the MS cohort. Red in the heatmap denotes individual subjects with the value of the individual ratio greater than the value of the ratio in all subjects within the CTRL cohort. Green denotes individual subjects with the value of the individual ratio less than or equal to the highest ratio value in all subjects within the CTRL cohort. **b**. Results from inputting independent CIS➔MS subjects into the ratioscore algorithm. Click here for file

Additional file 3: Figure S3 Ratios making up the ratioscore that discriminate MS from OND-NI or OND-I. **a**. Optimum ratios to discriminate MS from OND-I. **b**. Results for individual CIS ➔MS subjects using the MS : OND-I ratioscore. **c**. Optimum ratios to discriminate MS from OND-NI. **d**. Results for individual CIS➔MS subjects using the MS : ONDNI ratioscore. Click here for file
