# Supplementary material for: Using biomarkers to predict progression from clinically isolated syndrome to multiple sclerosis
Source: J Clin Bioinforma. 2013 Oct 3;3:18. doi: 10.1186/2043-9113-3-18 (PMC3850501; doi:10.1186/2043-9113-3-18)
Supplement: Additional file 1: Figure S1 — Flow chart describing sample collection and processing, data generation, and methods of data analysis. [file 2043-9113-3-18-S1.pdf]

SAMPLE COLLECTION

↓

RNA PURIFICATION

↓

cDNA SYNTHESIS

↓

LOAD TLDA PLATES & RT-PCR

↓

CONVERT C_T TO LINEAR EXPRESSION VALUES, $=2^{(40-CT)}$

↓

IDENTIFY DISCRIMINATORY SINGLE EXPRESSION RATIOS; eg. GENE A/GENE B, WHERE THE GREATEST NUMBER OF SUBJECT RATIO SCORES ARE HIGHER THAN THE HIGHEST OR SECOND HIGHEST RATIO SCOREIN THE COMPARATOR SUBJECT GROUP

↓

ESTABLISH A POINT SYSTEM: ONE POINT IS AWARDED TO A SUBJECT FOR EACH EXPRESSION RATIO WHERE IT ACHIEVES A HIGHER SCORE THAN THE HIGHEST OR SECOND HIGHEST RATIO SCORE IN THE COMPARATOR SUBJECT GROUP

↓

PERMUTATION TESTING TO IDENTIFY COMBINATION OF RATIOS, RATIOSCORE, THAT BEST DISCRIMINATES ALL SUBJECTS IN THE TEST GROUP FROM ALL SUBJECTS IN THE COMPARATOR GROUP

↓

TRAIN AND VALIDATE SVM.

↓

EVALUATE PREDICTIVE ACCURACY WITH INDEPENDENT CASE COHORT

Supplementary Figure 1. Flow chart describing sample collection and processing, data generation, and methods of data analysis.
