# Supplementary material for: Using biomarkers to predict progression from clinically isolated syndrome to multiple sclerosis
Source: J Clin Bioinforma. 2013 Oct 3;3:18. doi: 10.1186/2043-9113-3-18 (PMC3850501; doi:10.1186/2043-9113-3-18)
Supplement: Additional file 2: Figure S2 — a. Ability of the ratioscore method to discriminate between MS and combined CTRL plus OND subjects. Columns represent individual ratios. Rows represent individual subjects within the MS cohort. Red in the heatmap denotes individual subjects with the value of the individual ratio greater than the value of the ratio in all subjects within the CTRL cohort. Green denotes individual subjects with the value of the individual ratio less than or equal to the highest ratio value in all subjects within the CTRL cohort. b. Results from inputting independent CIS➔MS subjects into the ratioscore algorithm. [file 2043-9113-3-18-S2.pdf]

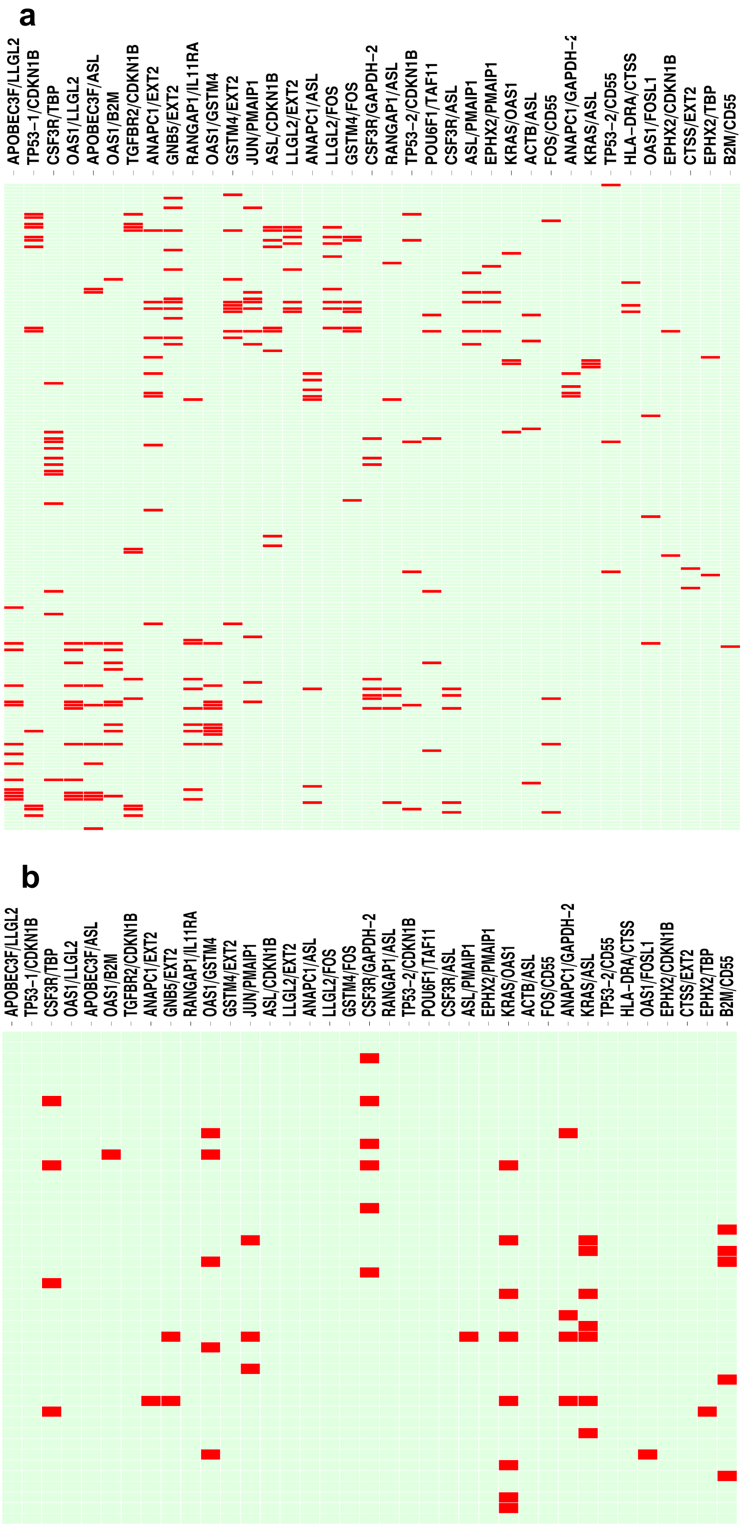

Supplementary Figure 2. **a**. Ability of the ratioscore method to discriminate between MS and combined CTRL plus OND subjects. Columns represent individual ratios. Rows represent individual subjects within the MS cohort. Red in the heatmap denotes individual subjects with the value of the individual ratio greater than the value of the ratio in all subjects within the CTRL cohort. Green denotes individual subjects with the value of the individual ratio less than or equal to the highest ratio value in all subjects within the CTRL cohort. **b**. Results from inputting independent CIS➔MS subjects into the ratioscore algorithm.
