# Supplementary material for: Using biomarkers to predict progression from clinically isolated syndrome to multiple sclerosis
Source: J Clin Bioinforma. 2013 Oct 3;3:18. doi: 10.1186/2043-9113-3-18 (PMC3850501; doi:10.1186/2043-9113-3-18)
Supplement: Additional file 3: Figure S3 — Ratios making up the ratioscore that discriminate MS from OND-NI or OND-I. a. Optimum ratios to discriminate MS from OND-I. b. Results for individual CIS ➔MS subjects using the MS : OND-I ratioscore. c. Optimum ratios to discriminate MS from OND-NI. d. Results for individual CIS➔MS subjects using the MS : ONDNI ratioscore. [file 2043-9113-3-18-S3.pdf]

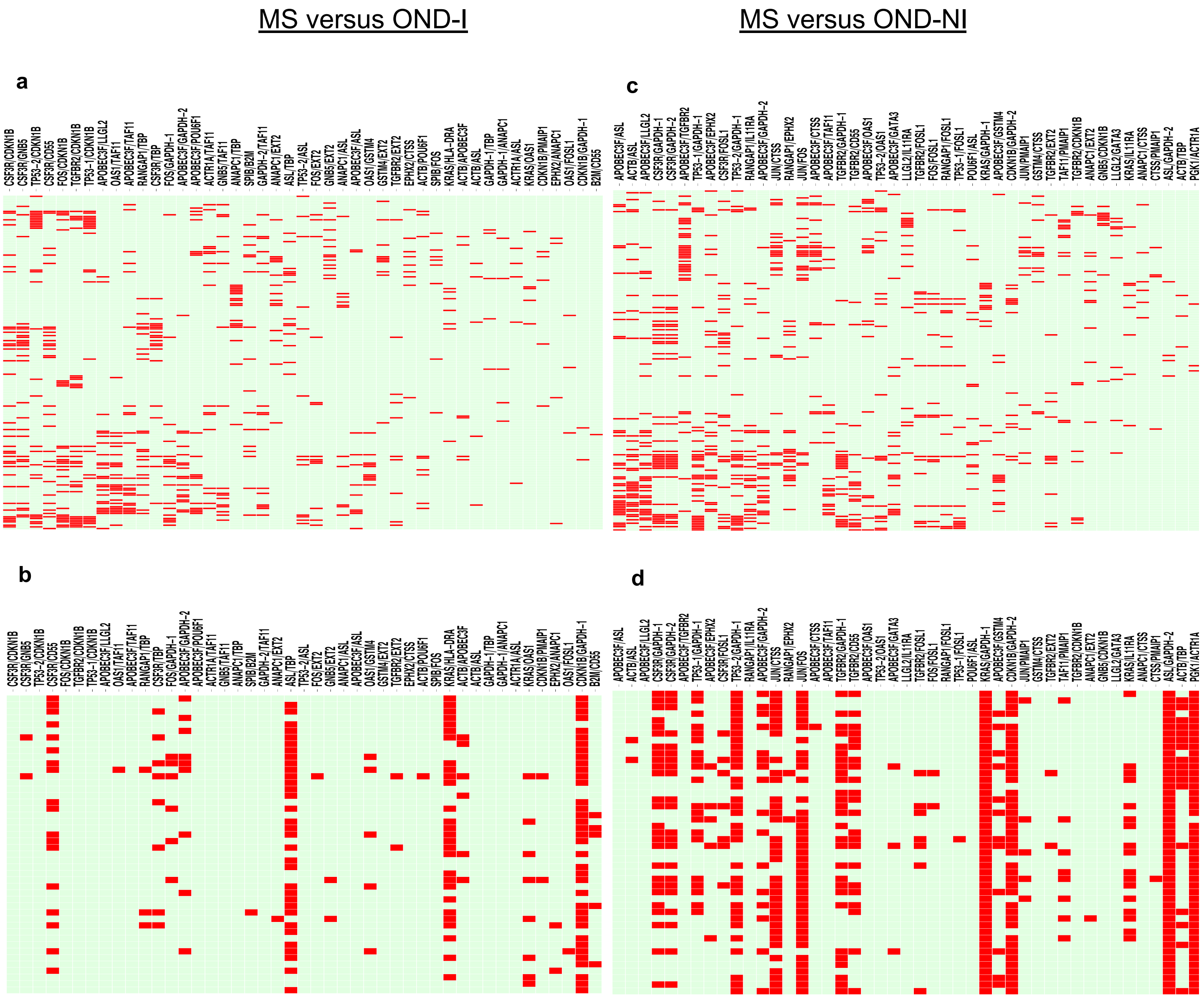

Supplementary Fig. 3. Ratios making up the ratioscore that discriminate MS from OND-NI or OND-I. **a**. Optimum ratios to discriminate MS from OND-I. **b**. Results for individual CIS ➔ MS subjects using the MS : OND-I ratioscore. **c**. Optimum ratios to discriminate MS from OND-NI. **d**. Results for individual CIS ➔MS subjects using the MS : OND-NI ratioscore.
